# Recent Progress in Dentistry

**Published:** 1886-06

**Authors:** William Herbert Rollins


ARTICLE IV.

### RECENT PROGRESS IN DENTISTRY.

BY WILLIAM HERBERT ROLLINS.

### A.

### *Altered Salivary Secretion.*[1]

From each parotid duct there exuded a thick purulent fluid, which to the naked eye, as well as to microscopic examination, presented all the chararters of "laudable" pus; the mouth was somewhat dry, and the whole mucous membrane had a curious reticulated look; the pus about the orifices of the ducts was free from odor and did not contain organisms, but the breath was unpleasaut. The pus was strongly alkaline. The secretion began after an attack of typhoid fever and had existed for twenty years. The other oral secretions were normal. The patient's health was excellent, her digestion apparently perfect. The case is of great interest as showing that health can be maintained without parotid secretion, and while daily swallowing large amounts of pus.

### *Epiphora and slight Ectropion cured by the extraction of a tooth.*[2]

There was a fistulous opening about half an inch before the inner canthus of the right eye, and well marked epiphora, as well as slight ectropion on that side. On an examination no canine could be found. An incision into the jaw over the position usually occupied by this tooth revealed the presence of a buried canine root. When this was extracted all the symptoms disappeared gradually.

1Tomes in Journ. Brit. Dent. Assn., p. 465.

2J. M. Ackland, in Journ. Brit. Dent. Assn., p. 467.

*Calcification of the Tooth Pulps caused by Neuralgia of the Trigeminal Nerve.*[3]

The patient had suffered with violent toothache for five months. Seven teeth had been extracted during this time without any relief. The extracted teeth were only slightly carious and had been filled. As the woman was only twenty, he did not consider that the new formations of dentine which was found in all the extracted teeth could have been physiological, as they might have been in an old person. Nor did the disease seem one originating in the teeth, for it extended to so many. In seeking for a cause elsewhere it was found that the trigeminal nerve was affected. The fontal, the supra, and infraorbital, and the zygomatic points were all very tender to pressure. The diagnosis was neuralgia of the second and third branches of the trigeminal nerve. The patient was anæmic, and of a family subjected to nervous diseases. The treatment, which was successful consisted of Fowler's solution, bromide of potash, and sulphate of iron. This case is interestering as these pulp ossifications are undoubtedly on the increase, and there is a tendency to consider them local in origin.

*Necrosis of Alveolar Ridge after Measles.*[4]

Three cases are reported, but the details are not given.

*Crowded Arches the cause of Riggs' Disease.*[5]

The writer considers that lateral compression is the predisposing cause of the wasting of the alveolar processes. The jaws are not large enough to allow the teeth to asume proper positions. He think that fungi are the intermediate cause of the inflammatory process.

*Epilepsy from Dental Caries.*[6]

Two cases are given in which the epileptic seizures disappeared immediately after the extraction of a carious tooth.

---

3 Spoffella in Brit. Journ. of Dent. Science, October, 1885.
4 Coterell in Brit. Journal of Dent. Science, p. 132, 1886.
5 Bate in South. Dent. Journ., p. 64, 1886.
6 Leibert in Brit. Journ. of Dent. Science. p. 116, 1886.

### *Lagophthalmus due to Dental Irritation.*[7]

The left eyelid was so drawn up that the white showed above the iris. The second and third left molars were extracted because decayed. This relieved a persistent neuralgia with which the patient had suffered, but had no effect upon the eyelid. A year after it was decided to remove a filling in the left upper third molar. An exposure of the pulp was found. The tooth was extracted, and as a result the trouble in the eye gradually got well, having entirely disappeared in six months.

### *Effect of Mental Overwork upon the Teeth.*[8]

Among the hard-worked pupils of the Paris public schools, the teeth become deteriorated in a few weeks after entry. The second dentition is often premature. These observations confirm the statements of Dr. J. L. Williams, who has given great attention to this subject. He has shown that any mental strain shows itself upon the teeth in a short time, both in increased decay, as well as in increased sensibility of the dentine. Dr. D. M. Parker has reported that these same changes are always apparent in men who are in training for athletic trials. As there is not the slightest doubt of the accuracy of these observations, they show that these are matters which demand serious consideration from educators.

### *Spinal Irritation from the Teeth.*[9]

The patient, a girl of fourteen, had been under treatment for two years for reflex spinal irritation which was considered hysterical. An examination of the mouth showed that the bicuspid teeth were low down and wedged between the molars. The molars were extracted, and it was then found that the pulps were exposed. This exposure was thought to be due to absorption caused by the bicuspids. The patient recovered at once.

---

7 Dent. Cosmos, p. 123, 1886.
8 V. Gallippe in Brit. Journ. of Dent. Science, p. 995, 1885.
9 Redman in Brit. Journ. of Dent. Science. p. 715, 1885.

*Epistaxis caused by a Tooth.*[10]

There was severe hæmorrhage from the right nostril at intervals for several days. The upper right lateral was extracted; the blood flowed profusely from the nose but soon subsided and the bleeding did not return.

B.

*Alveolar Dental Membrane.*[11]

This membrane consists of two distinct layers, developed from separate centres. The outer layer of fibrous connective tissue is the periosteum of the bone, the inner a layer of fine cells, originating in the dental follicle is the true root membrane.

*Is a Transplanted or Replanted Tooth Pulpless?*[12]

He relates two cases in which in regulating, it became necessary to rotate an upper lateral incisor. The ordinary means failing, the tooth was extracted and replaced in the proper position. These teeth have never discolored, and are as sensitive to heat and cold as the other teeth in the same mouth. This he thinks proves that the pulps are living.

Two similar cases have come under my observation in which perfectly healthy teeth in young persons having been entirely separated from the jaws by blows were replaced and to all appearance have healthy pulps; and when afterward they decayed, the dentine was sensitive.

*A new Method of Recording the movements of the Soft Palate.*[13]

A straight rod is passed through the nose from before backward, in the living subject. In this position it is not influenced by the movements of the soft palate; but if the end of the rod which remains without the nostril is raised

---

10 Johnston in Dental Cosmos, November, 1885.
11 Ingersol in South. Dent. Journ. p. 415, 1885.
12 Underwood in Brit. Journal of Dent. Science. p. 30, 1886.
13 Allen in Ohio State Journ. of Dent. Science. p. 551, 1885.

so that it comes in contact with the anterior border of the nostril, the pharyngeal end will lie in such a position as to receive a decided motion when the soft palate is raised. Supporting this rod from the forehead and recording its motions on carbonized paper gives a means of studying the motions of the soft palate in deglutition, respiration, coughing, hawking and snuffing, etc.

### *Progression in Teeth.*

Human teeth have a tendency to move forward. This is seen clearly under several conditions. If a molar is extracted the teeth behind the gap move forward so that the space is almost entirely closed from this movement. If a tooth is extracted in regulating to make room and we wish to draw back the teeth in front of the gap, we shall find it necessary to have the frame from which the tension is exerted, extend around several teeth behind the space, for if the frame is attached to only one tooth on each side of the space the tooth behind will move forward much more than the other will move back. This is true even where the tooth behind the space is large and firmly implanted by means of three roots, as in a molar. The following case illustrates this: A canine tooth erupted behind the lateral incisor. There was much pain of a neuralgic character which I could account for only on the supposition that this tooth in some way acted as an irritant to the first bicuspid in which the pain was located. The bicuspid was therefore taken out and an attempt made to draw the canine back. A frame extending around all the three molars and second bicuspid was made and used as the fixed point from which tension on the canine was exerted, but the canine remained stationary while all the teeth behind began to move forward. It was found necessary to have a plate attached to all the available teeth in the mouth to prevent the teeth from moving forward before the canine moved back. This is an extreme case as the root of a canine is deeply implanted, but it serves to illustrate this tendency in teeth which I am certain exists. We see it plainly in some of the lower animals as in the elephant, who has a constant progression of the molars whereby the masticating surfaces are kept constantly renewed.

C

*Rapid Separators.*

Numerous attempts have been made, from time to time, to produce an instrument for quickly separating the teeth, to prepare for filling. None of these attempts were succesful. Recently, Dr. Bogue has devised a series of these instruments, which are of great value, as with them, enough space can be got in a few minutes to enable filling to be done without previous wedging, in the ordinary slow way.

*Danger from small Artificial Plates.*

Several cases of death from swallowing artificial plate, are mentioned in the journals for the past year. This shows how important it is to warn patients not to sleep with such appliances in their mouths.

*An All-Porcelain Crown.*[14]

The crown consists of a piece of keramic body baked to the shape of the crown, which has been lost by decay. Wires projecting above the roots, into which they are firmly fixed, are the means of holding the crown in position, as they project into a hollow in the crown, being fastened there with cement.

*A Band Matrix.*[15]

The matrix consists of a narrow and thin band of steel of sufficient length to go three-quarters around a tooth. To use the matrix, it is bent around the tooth to be filled, and held in position by a small clamp, the ends of which go into holes in the ends of the band. By turning the screw in the clamp, the band is drawn tightly against the tooth, and held there.

*Fractured Teeth.*

The usual explanation of a fracture in a tooth, is to say

---

14 Howland in Ind. Pract. p. 187, 1886.

15 Guilford in Dent. Cosmos, p. 137. 1886.

that the tooth has received a blow. Redman[16] reports a case in which there was no history of a blow, nor, indeed, had the tooth any antagonistic tooth, so that it seems impossible to consider the fracture traumatic. In this case, the first symptom was severe pain, which lasted for two days. Then the tooth became loose, and was found, by the dentist, to be split in halves lengthwise.

*An Impacted Tooth from a Fall.*[17]

The patient, falling from a horse, drove the right upper lateral into the jaw, so that it could not be seen from the front. The tooth was grasped with thin forceps, drawn into place. and held with splints for four days, when it was firm enough to do without further treatment.

*A new operation for Salivary Fistula.*[18]

The fistula, which was caused by a stab, opened on the cheek, and had no communication with the mouth. Two needles, threaded on one fairly-strong piece of silk, were successfully passed through the fistula into the mouth, piercing the buccal membrane one-quarter of an inch apart; the ends were firmly tied, inclosing a portion of the duct, and the surrounding tissues. The internal wound healed in a few weeks, and the saliva found its way into the mouth, as usual.

*Accidents in Tooth Extraction.*

At the December meeting of the Odont. Society of Great Britain, MacCormac related a case in which, during the extraction of a tooth, the forceps broke, and one blade disappeared. The patient immediately became "black" in the face, and almost suffocated. These symptons gradually disappeared, and at the end of six weeks, there was no great cause for anxiety, though she suffered from pain on the right side of the sternum, spasmodic cough and bloodly expectoration; so it was decided to open wind-pipe.

---

16 Brit. Journ. of Dental Science. p. 715, 1885.

17 Tomes in Brit. Jour. of Dental Science, January 15, 1886.

18 Hodgson in Dent. Cosmos, November 1885.

Though this, in opening the end, the broken blade could be felt,five inches below, in the right bronchus. The fragment was removed, the patient making a good recovery. As the forceps were made by Evrard, the most distinguished maker who has ever lived, there is no reason to think that they were not as perfect as possible. The case, therefore, shows the importance of guarding the throat in tooth extraction. A case occurring in my practice, shows one other danger, which has never been mentioned. In an attempt to extract an upper bicuspid, the forceps slipped, and closing just as it reach the lip, it took a piece of this between the blades and severed it entirely, thus leaving a permanent scar. As there is no use in having the blades of forceps with sharp-cutting edges, which come in contact, this very common form ought to be discarded.

*Preparing for the Eruption of the First Permanent Molars.*

These teeth usually erupt when a child is six years old. They almost always decay, either during eruption or shortly afterward. Until the eruption of the bicuspids, five years later, the anterior surfaces of these teeth are in contact with the first, or temporary molars. It is a well-established fact, that contact between teeth will result in decay in most mouths. As the temporary molars are only to remain in the mouth for a few years, there is no objection to so cutting away the posterior surfaces of these teeth, so as to give as small a contact as possible between them and the first permanent molars. A dentist should always see a child shortly after the age of five years, in order that this treatment may be carried out before the permanent teeth erupt, because after this, it is difficult to properly do the extensive cutting without injury to the permanent tooth, whose enamel is always soft at first. If it were possible to grind away the backs of the temporary molars, and thus prevent the permanent ones from ever touching them, the danger of decay would be slight, but there is a peculiar tendency in teeth, which has not been properly explained, or, so far as I am aware, noticed; this is the tendency which they show to move toward the front of the mouth. I shall speak of this later, under the head of "Progression in Teeth." It is due to this tendency, that it is not practical, in most cases, to maintain perfectly free spaces in the positions named.

The best one can do, is to grind the backs of the temporary teeth at small points near the grinding surfaces, for here, contact will do the least harm: first because the enamel is strongest there; and second, because, if decay should begin, it can be more easily managed than at any other point on the approximal surfaces. Without a figure it is not easy to explain the shape this preventive trimming should make the teeth.

*Fatal Hæmorrhage from Tooth Extraction.*[19]

Patient, aged twenty two, was admitted to the hospital for chronic synovitis of right knee, caused by a blow. Ten weeks after this, an aching lower molar was extracted. The bleeding ceased in a few minutes, but soon returned so perfuriously, that it was necessary to plug the socket. The bleeding continued, without interruption for seven days, when the patient died. Just before death, the temperature had risen to 102.6° F. All known styptics were tried. Stimulants were given at short intervals. As a similar case, in which transfusion was used, was given in my report in 1884, in which the patient recovered, after he had become practically dead, it seems worth while to speak of this again as this treatment did not seem to have occurred to the medical men who had this case in charge.—*Boston Med. and Surg. Journal.*

---

19 Albert, Dental Record. p. 85, 1886.

